# The effect of long-term administration of green tea catechins on aging-related cardiac diastolic dysfunction and decline of troponin I

**DOI:** 10.1016/j.gendis.2024.101284

**Published:** 2024-04-03

**Authors:** Junjun Quan, Zhongli Jia, Lingjuan Liu, Jie Tian

**Affiliations:** a Department of Cardiology, Children's Hospital of Chongqing Medical University, National Clinical Research Center for Child Health and Disorders, Ministry of Education Key Laboratory of Child Development and Disorders, Chongqing 400014, China; b Chongqing Key Laboratory of Structural Birth Defect and Reconstruction, Chongqing 400014, China; c Department of Pediatrics, The People's Hospital of Leshan, Leshan, Sichuan 614000, China

**Keywords:** Aging, Cardiac diastolic dysfunction, Cardiac troponin I, Epigallocatechin gallate, Histone deacetylase 1

## Abstract

Aging is an independent risk factor for cardiovascular diseases. Cardiac diastolic dysfunction (CDD), ultimately leading to heart failure with preserved ejection fraction (HFpEF), is prevalent among older individuals. Although therapeutics have made great progress, preventive strategies remain unmet medical needs. Green tea catechins have been shown to be effective in improving aging-related cardiovascular and cerebral disorders in animal models and patients. However, little attention has been paid to whether long-term administration of epigallocatechin gallate (EGCG), the major bioactive ingredient of green tea catechins, could prevent the onset and progression of CDD. In this study, 12-month-old female mice were orally administered 50, 100 and 200 mg EGCG mixed with drinking water for 6 months. Aged mice (18 months old) exhibited the major features of HFpEF, including CDD with pEF, cardiac fibrosis, increased cardiomyocyte apoptosis, and mitochondrial damages, as well as elevated A/B-type natriuretic peptide. Cardiac troponin I (cTnI) expression was also reduced. Long-term administration of 100 or 200 mg EGCG prevented aging-related CDD and exercise capacity decline, along with alleviating myocardial apoptosis and mitochondria damage. The transcription and protein expression of cTnI were increased, which might be achieved by inhibiting the expression and activity of histone deacetylase 1 (HDAC1), and reducing its binding level near cTnI's promoter, thereby elevating acetylated histone 3 (AcH3) and acetylated lysine 9 on histone H3 (AcH3K9) in the aged mice. We provide a novel insight that long-term administration of EGCG is a potentially effective strategy in preventing aging-related CDD and cTnI expression decline.

## Introduction

Aging is an independent risk factor for cardiovascular diseases, especially for heart failure (HF). Cardiac diastolic dysfunction (CDD) underlies HF with preserved ejection fraction (HFpEF) and is generally considered an aging-related condition. CDD is characterized by impaired relaxation with normal systolic function, however, it impacts approximately 3 million people in the United States and up to 32 million people worldwide.^1^^,^^2^ As reported, CDD has been one of the leading causes of cardiovascular mortality in the elderly.^3^^,^^4^ Patients with CDD have a very poor prognosis with more than 50% deaths 5 years after diagnosis.^5^ Nevertheless, effective treatments to improve clinical outcomes and long-term survival in elderly patients with CDD are scarce. The underlying mechanisms are still being explored.^6^, ^7^, ^8^

Cardiac troponin I (cTnI) plays a key role in regulating diastolic function. Changes including deficiency and mutations in cTnI are associated with CDD.^9^^,^^10^ In elderly human hearts, the cytoplasmic concentration of cTnI is decreased in the left ventricular (LV) myocardium.^11^ Aging-related CDD is also associated with decreased cTnI expression caused by abnormal histone acetylation modification.^10^ Epigallocatechin gallate (EGCG), the main green tea catechin extracted from *Camellia sinensis*, has emerged as a mediator of cardiovascular health.^12^ Cardioprotective effects of EGCG have been documented in several clinical studies.^13^^,^^14^ In our recent clinical trial, cardiac functions and HF symptoms were significantly improved in cardiomyopathy patients who received EGCG capsules daily for 12 months.^15^ Green tea intake was also related to enhanced blood vessel function and lower heart disease risk.^12^ Several studies have shown that EGCG, as a histone acetylation modification regulator, could alter gene expression via inhibiting histone deacetylase 1 (HDAC1) and regulate organ functions.^16^^,^^17^ So far, little attention has been paid to whether specific long-term intervention with EGCG could prevent the onset and progression of CDD and cTnI expression decline. In this study, we investigated the effects of three doses of EGCG on CDD and the underlying mechanisms.

## Materials and methods

### Animals

**Figure 1:**
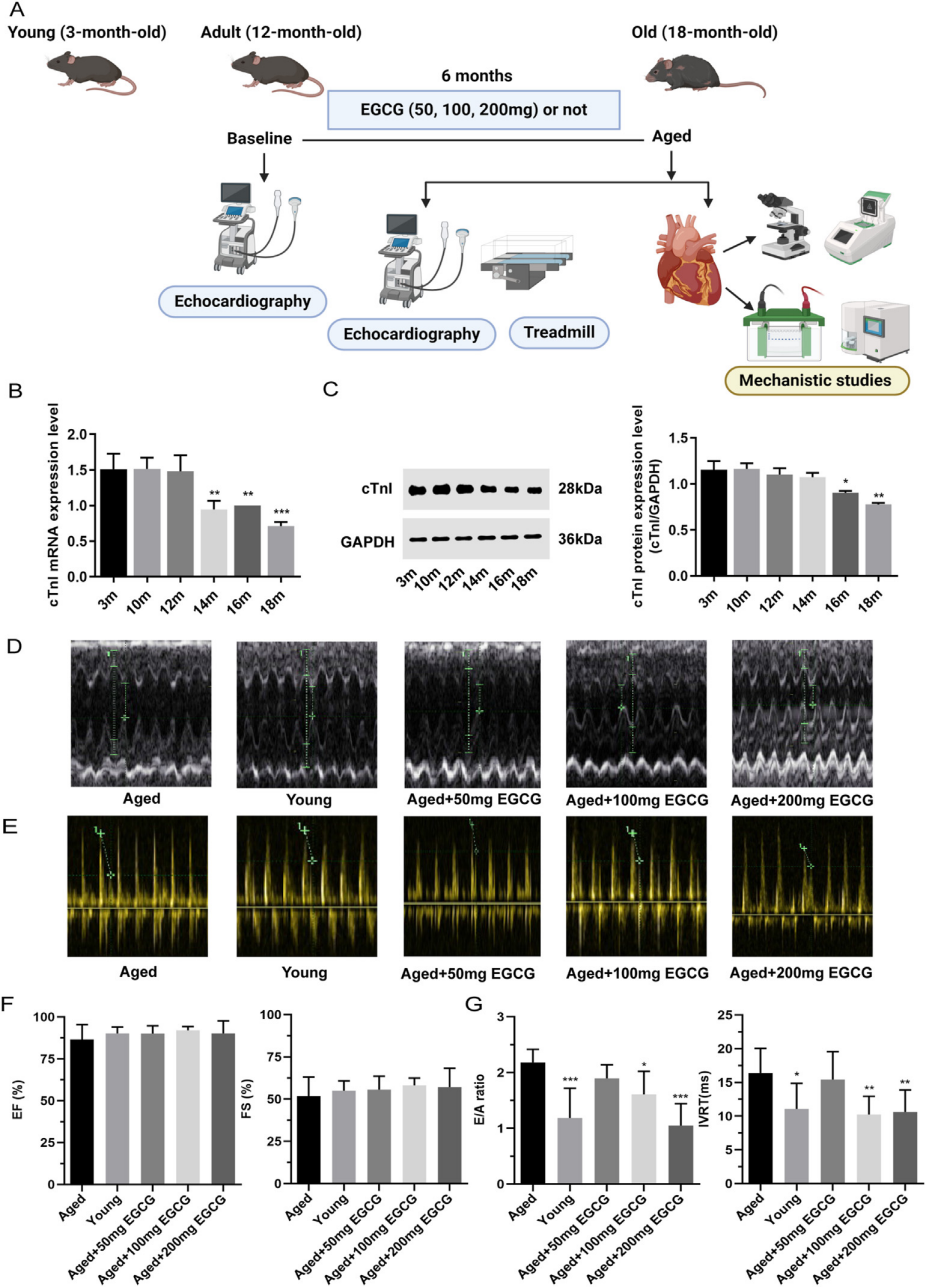
Long-term administration with EGCG prevents diastolic dysfunction later in life in the aged mice. **(A)** Diagram of the study design: mice were subjected to transthoracic echocardiography for assessment of cardiac functions, to treadmill test for evaluation of exercise ability, and to mechanistic studies. **(B, C)** Changes in transcription and protein expression of cardiac troponin I (cTnI) at different ages. **(D)** Representative M-mode echocardiographic images acquired from LV short-axis views. **(E)** Representative Doppler echocardiographic tracings. **(F, G)** Quantitative measurements of cardiac functions including ejection fraction (EF), fractional shortening (FS), the ratio of early ventricular filling to late ventricular filling caused by atrial contraction (E/A), and isovolumetric relaxation time (IVRT) (*n* = 8–10). All results are represented as mean ± standard deviation. ^∗^*P* < 0.05, ^∗∗^*P* < 0.01, ^∗∗∗^*P* < 0.001.

Female C57BL/6 mice were purchased from the Animal Center of Chongqing Medical University. All studies were carried out in strict accordance with the recommendations approved by Chongqing Medical University (Chongqing, China). All mice were housed in five per cage in the controlled environment with a 12 h strict light/dark cycle at 23 ± 1 °C and fed a standard diet from 12 to 18 months of age for 6-month administrations. Mice had free access to drinking water and food. As summarized in Figure 1A, forty 12-month-old mice were randomized into four groups: aged, 50 mg EGCG (50 mg/kg/day), 100 mg EGCG (100 mg/kg/day), and 200 mg EGCG groups (200 mg/kg/day), containing 10 animals each. EGCG-treated mice were orally administered EGCG (purity: 99%, Selleck, USA) mixed with drinking water. Moreover, the aged mice were orally administered ordinary drinking water without an intervention agent. Mice aged 3 months were assigned as young control group (*n* = 10). Drinking water containing EGCG was freshly prepared at about 5:00 p.m. every day at room temperature for EGCG-treated groups, and water left in the bottle was measured to determine the amounts consumed and then replaced. All mice were subjected to transthoracic echocardiography for assessment of cardiac functions at the age of 12 and 18 months, as well as a treadmill test. Heart tissues were harvested for histological analysis and determination of cardiac proteins, histone acetylation modification, and myocardial apoptosis and fibrosis.

### Echocardiography

Mice were subjected to conventional transthoracic echocardiography and Doppler analyses using GE Vivid E9 color Doppler ultrasound system (USA) equipped with a high-frequency probe (11 MHz) before (12 months old) and after (18 months old) interventions. Experimental animals were anesthetized with 2% isoflurane for 3–5 min and then maintained with 1%–1.5% during data collection. Ventricular dimension, septal and ventricular wall thickness at end-diastole and end-systole were measured to calculate systolic function using standard M-mode images under a short axis view. The mitral blood inflow and diastolic function were evaluated using the pulse-wave Doppler. Additionally, the following parameters were measured as well: early ventricular filling (E) to late (A) ventricular filling caused by atrial contraction (E/A), isovolumetric relaxation time (IVRT), and isovolumetric contraction time (IVCT). All data were analyzed using *in vivo* echocardiography report software. Both the echocardiography operator and data analyzer were blinded to the groups.

### Treadmill test

A treadmill test with a six-lane was used to assess exercise capacity, as previously described.^18^ After one-week training, the exhaustion test was performed, which comprised 3 exercise tests on the treadmill for consecutive days. All mice received a 1.2-mA shock if they stopped running and slid onto an electric grid placed at the rear end of the treadmill. An exercise test was started at a speed of 10 m/min on a 5° incline. The speed was increased by 1 m/min every 3 min until the mice fatigued, that is, the animals could no longer keep pace with the belt, and at this point, the running distance was recorded.

### Heart tissue harvesting

Mice at 3, 10, 12, 14, 16, and 18 months of age were anesthetized with carbon dioxide and sacrificed. Heart tissues were washed with phosphate-buffered saline and stored at −80 °C. After administrations, heart tissues from young (3 months old), aged (18 months old), and EGCG groups were harvested.

### Morphological observations

LV samples were prepared for the transmission electron microscope (TEM), cut into small pieces about 1 mm^3^, fixed immediately with 3% glutaraldehyde, and sent to the TEM center of Chongqing Medical University. All samples were sliced by ultramicrotome (Leica, German) and observed using a Transmission Electron Microscope (Hitachi, Japan). Formalin-fixed and paraffin-embedded tissues were cut into sections and stained with hematoxylin-eosin (HE). Cardiac fibrosis was observed using Masson's trichrome and collagen volume fraction (CVF) was calculated by NIH ImageJ Software as the ratio of fibrosis to total tissue area.

### Terminal deoxynucleotidyl transferase dUTP nick end-labeling (TUNEL)

TUNEL assay was carried out according to the manufacturer's instructions (Roche, Germany) for observing cardiomyocyte apoptosis. The nuclei were marked in blue, while TUNEL-positive cells were stained in green. The fluorescence intensity of apoptotic cardiomyocytes was documented by a fluorescence microscope (Nikon, Japan). The apoptotic index was estimated as the ratio of the number of apoptotic cells to the total number counted.

### Mouse primary cardiomyocyte culture, transfection and treatment

Primary cardiomyocyte culture from neonatal C57BL/6 mice was performed as previously described.^19^ The collected cells were divided into Blank, DMSO, NC, HDAC1 overexpression, and HDAC1 overexpression + EGCG groups. Cardiomyocytes were transfected with empty vector or overexpression HDAC1 adenovirus. In HDAC1 overexpression + EGCG group, cardiomyocytes were treated with EGCG (40 μM) right after transfection with HDAC1 overexpression adenovirus. Cells were collected 24 h after administrations for subsequent analysis.

### Quantitative real-time PCR

Total RNA was isolated from heart tissues using an RNA Extraction kit (Bioteck, China). Single-strand cDNA synthesis was performed using oligo dT-Adaptor primers and an AMV reverse transcriptase kit (TaKaRa, Japan). cDNA amplification was performed according to a quantitative real-time PCR assay employing a SYBR Green RealMasterMix kit (Tiangen, China). β-actin was used as a housekeeping gene to normalize transcription levels of the genes of interest. Genes of interest, including cTnI, A/B-type natriuretic peptide (ANP and BNP), HDAC1/2/3, sarcoplasmic reticulum Ca^2+^-ATPase (SERCA2a), sarcalumenin (SAR), and control were detected with the corresponding gene-specific pairs of primers, as detailed in Table S1.

### Western blotting

Western blotting was performed in accordance with previous protocol.^20^ The homogenized samples were obtained using ice-cold RIPA buffer (Beyotime, China) containing protease and phosphatase inhibitors, and the protein concentration was determined using the Bradford Assay (Thermo Fisher, USA). Proteins of interest and control transferred to the PDVF membrane were analyzed using primary antibodies against cTnI (Abcam, USA), HDAC1 (CST, USA), or GAPDH (Arigo, China). The membranes with proteins were incubated with corresponding HRP-labeled secondary antibodies (Beyotime, China). Immunoblots were visualized by enhanced chemiluminescence luminal reagent (Mishushengwu, China) through Imaging System (Bio-Rad, USA) and quantitative analyses were acquired using Quantity One software (Bio-Rad, USA).

### Chromatin immunoprecipitation

Chromatin was collected using the Chromatin Extraction Kit (Abcam, England) and immunoprecipitated in accordance with the instructions of the chromatin immunoprecipitation (ChIP) one-step kit (Abcam, England). The chromatin was fragmented into 200–1000 bp DNA fragments by ultrasonication. Protein-DNA complexes were incubated using antibodies against acetylated histone 3 (AcH3, Abcam, England), acetylated lysine 9 on histone H3 (AcH3K9, Abcam, England), acetylated lysine 27 on histone H3 (AcH3K27, Abcam, England), HDAC1 (Cell Signaling Technology, USA), histone deacetylase 2 (HDAC2, Cell Signaling Technology, USA), histone deacetylase 3 (HDAC3, Cell Signaling Technology, USA), GATA4 (Abcam, England) or MEF2c (Abcam, England), alongside non-immune IgG (as a negative control) and RNA polymerase II (as a positive control). The primer sequences targeting the −1000 bp upstream regions of the cTnI promoter were listed in Table S2.

### HDAC1 activity assay

HDAC1 activity was detected using an HDAC1 Activity Assay Kit (Biovision, USA). Proteins were extracted as per the manufacturer's instructions and the protein concentrations were tested using the Bradford Assay (Thermo Fisher, USA). A total of 50 μg protein for each immunoprecipitation was added with 6 μL HDAC1 antibody in sample groups or 6 μL IgG in control groups, followed by incubation at 4 °C overnight. After incubation, the protein-A/G bead slurry of 25 μL was added and incubated at 4 °C for 1 h. The beads were collected by centrifugation and wash. For each reaction, sample and control groups were mixed and incubated with Reaction Mix containing HDAC Assay Buffer and HDAC Substrate at 37 °C for 2 h. The Positive Control, Developer, and Standard Curve were prepared according to the manufacturer's instructions. Reaction supernatant of 100 μL was transferred to individual wells and the fluorescence value was read at Ex/Em = 380/500 nm. Then the AFC standard curve was gained. Sample HDAC Activity = (2 × B)/(T × S) = pmol/min/mg = mU. B was AFC amount from the Standard Curve (pmol). Sample dilution factor was 2. T represented reaction time (120 min) and S represented sample amount (50 × 10^−3^ mg).

### Statistical analysis

All data were presented and analyzed using SPSS 23.0 software (USA) and GraphPad Prism 8.0 (USA). The results were shown as mean ± standard deviation (SD) and analyzed by one-way ANOVA followed by Tukey's or Bonferoni's or Sidak's multiple comparison post hoc. At least three independent triplicated experiments were performed for each experimental set-up. *P* values less than 0.05 were considered statistically significant, indicated as ^∗^*P* < 0.05, ^∗∗^*P* < 0.01, and ^∗∗∗^*P* < 0.001.

## Results

### The expression of cTnI decreased gradually during cardiac aging

To identify the key temporal point of cTnI expression decline during cardiac aging, the transcript and protein levels were measured in mice at 3, 10, 12, 14, 16, and 18 months of age. As shown in Figure 1B and C, cTnI mRNA expression declined markedly at 14 months of age (14 m, 16 m, or 18 m *vs*. 12 m, *P* < 0.01, respectively), and its protein expression was decreased significantly at 16 months of age (16 m or 18 m *vs*. 12 m, *P* < 0.05, respectively). This study was aimed to investigate the preventive effects of EGCG on cardiac aging. Therefore, adult mice at 12 months of age were given EGCG for 6 months. As previously described, cTnI expression had not yet decreased at 12 months.

### Long-term administration of EGCG prevented CDD in the aged mice

Echocardiographic data showed that diastolic function was preserved at the age of 12 months (Table S3), along with equivalent IVRT and E/A ratio to the young mice, which also suggested EGCG interventions should be initiated at this age. We then assessed heart functions in the young and aged mice treated with or without EGCG (Table S3; Table 1). In the present study, the LV systolic function, as shown by pEF and fractional shortening, remained unchanged in the aged mice (18 months old), the same as in the young mice (*P* > 0.05 for each of the four aged groups *vs*. the young group; Fig. 1D, F and Table 1). However, a deteriorative and noticeable reduction of LV diastolic function was observed in the aged mice, as reflected by a significant increase of the E/A ratio (*P* < 0.001) and a prolongation of IVRT (*P* < 0.05) compared to the young mice (Fig. 1E, G and Table 1). Fortunately, long-term administration of 100 and 200 mg EGCG effectively prevented CDD: the E/A ratio was 0.74-fold and 0.48-fold lower, and IVRT was 0.79-fold and 0.65-fold lower in 100 and 200 mg EGCG-treated mice, respectively, as compared with the aged mice, but no significant improvement of both was observed after 50 mg EGCG administration (100 or 200 mg EGCG *vs*. the aged group: *P* < 0.05 for both; 50 mg EGCG *vs*. the aged group: *P* > 0.05 for both; Fig. 1E, G and Table 1).

**Table 1:** Cardiac functions after 6-month EGCG administration (18 months).

Parameters	Aged	Young	Aged+50 mg EGCG	Aged+100 mg EGCG	Aged+200 mg EGCG
IVSd (cm)	0.089 ± 0.020	0.086 ± 0.017	0.091 ± 0.010	0.089 ± 0.014	0.085 ± 0.008
LVIDd (cm)	0.305 ± 0.062	0.223 ± 0.046	0.271 ± 0.021	0.270 ± 0.030	0.269 ± 0.029
LVPWd (cm)	0.072 ± 0.025	0.075 ± 0.010	0.081 ± 0.010	0.080 ± 0.018	0.068 ± 0.005
LVIDs (cm)	0.153 ± 0.066	0.100 ± 0.025	0.119 ± 0.014	0.104 ± 0.023	0.116 ± 0.034
EDV (ml)	0.082 ± 0.050	0.030 ± 0.020∗∗	0.054 ± 0.013	0.055 ± 0.017	0.051 ± 0.014∗
ESV (ml)	0.012 ± 0.016	0.002 ± 0.004	0.004 ± 0.005	0.004 ± 0.005	0.003 ± 0.005
EF (%)	86.52 ± 8.97	90.09 ± 3.90	90.01 ± 4.69	92.06 ± 2.21	90.11 ± 7.62
FS (%)	51.76 ± 11.39	55.03 ± 5.75	55.59 ± 7.94	58.21 ± 4.28	57.09 ± 11.19
SV (ml)	0.066 ± 0.031	0.030 ± 0.020∗∗	0.049 ± 0.012	0.046 ± 0.015	0.047 ± 0.015
E/A	2.17 ± 0.24	1.19 ± 0.53∗∗∗	1.89 ± 0.24	1.61 ± 0.42∗	1.05 ± 0.39∗∗∗
IVRT (ms)	16.38 ± 3.68	11.07 ± 3.79∗	13.02 ± 5.36	10.24 ± 2.72∗∗	10.64 ± 3.25∗∗

A, late ventricular filling caused by atrial contraction; EDV, end-diastolic volume; EF, ejection fraction; ESV, end-systolic volume; FS, fractional shortening; IVSd, intraventricular septum at end-diastole; LVIDd, left ventricular internal dimensions at end-diastole; LVIDs, left ventricular internal dimensions at end-systole; LVPWd, left ventricular posterior wall thickness at end-diastole; All values are presented as mean ± SD. ^∗^*P* < 0.05, ^∗∗^*P* < 0.01, ^∗∗∗^*P* < 0.001; another four groups compared to 3 m, respectively.

**Figure 2:**
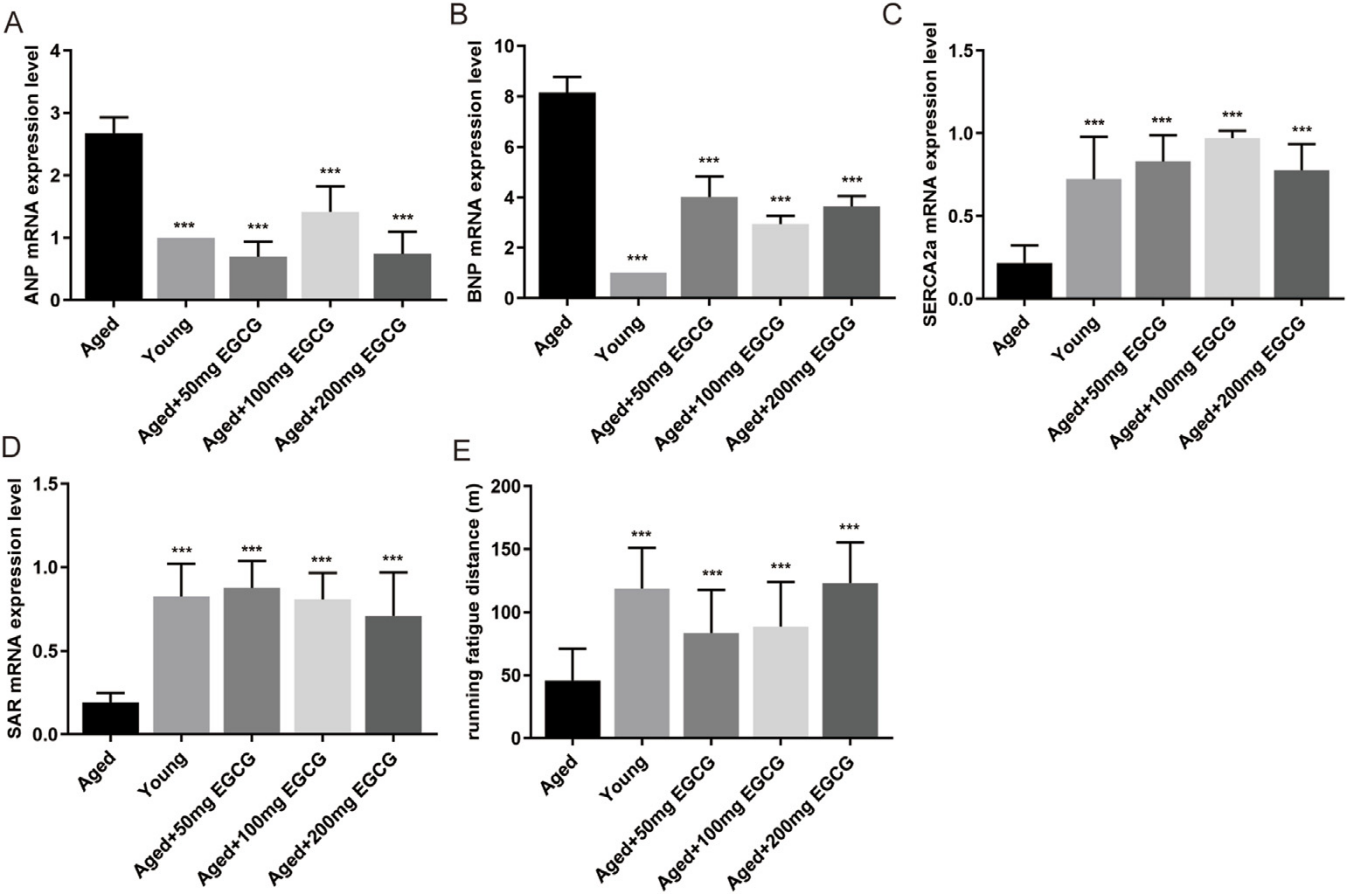
Long-term EGCG supplementation alleviates age-associated heart failure. **(A, B)** Gene expression of A-type natriuretic protein (ANP) and B-type natriuretic peptide (BNP). **(C, D)** Gene expression of sarcoplasmic reticulum Ca^2+^-ATPase (SERCA2a) and sarcalumenin (SAR). **(E)** Treadmill test for assessment of exercise capacity (*n* = 8–10). All results are represented as mean ± standard deviation. ^∗^*P* < 0.05, ^∗∗^*P* < 0.01, ^∗∗∗^*P* < 0.001.

Furthermore, ANP and BNP in LV myocardium, classical molecular markers of HF, were significantly enhanced in the aged mice, along with a marked reduction of heart aging markers SERCA2a and SAR compared to the young subjects (*P* < 0.001 for all parameters, Fig. 2A–D). Long-term EGCG intakes at all doses effectively improved cardiac aging phenotype as shown by a conspicuous reduction of HF markers and relieved the aging-related decreases of SERCA2a and SAR in the aged heart, compared with the aged mice (each of EGCG groups *vs*. the aged group: *P* < 0.001 for all parameters; Fig. 2A–D).

### Long-term administration of EGCG enhanced exercise performance in aged mice

A running exhaustion test was conducted to evaluate exercise capacity by treadmill test (Fig. 2E). Our findings showed that the average running fatigue distance of the aged mice was 54.52% lower than that of the young mice (*P* < 0.001). Concurrently, the aged mice supplemented with 50, 100, and 200 mg EGCG, respectively, exhibited increases in running fatigue distance by 37.01%, 31.36%, and 58.45% (each of EGCG groups *vs*. the aged group, *P* < 0.001), indicating EGCG intakes augmented exercise performance in the aged mice, with the most pronounced therapeutic effect observed in those treated with 200 mg EGCG.

### Long-term administration of EGCG repressed cardiomyocyte apoptosis and alleviated mitochondrial destruction in aged mice

**Figure 3:**
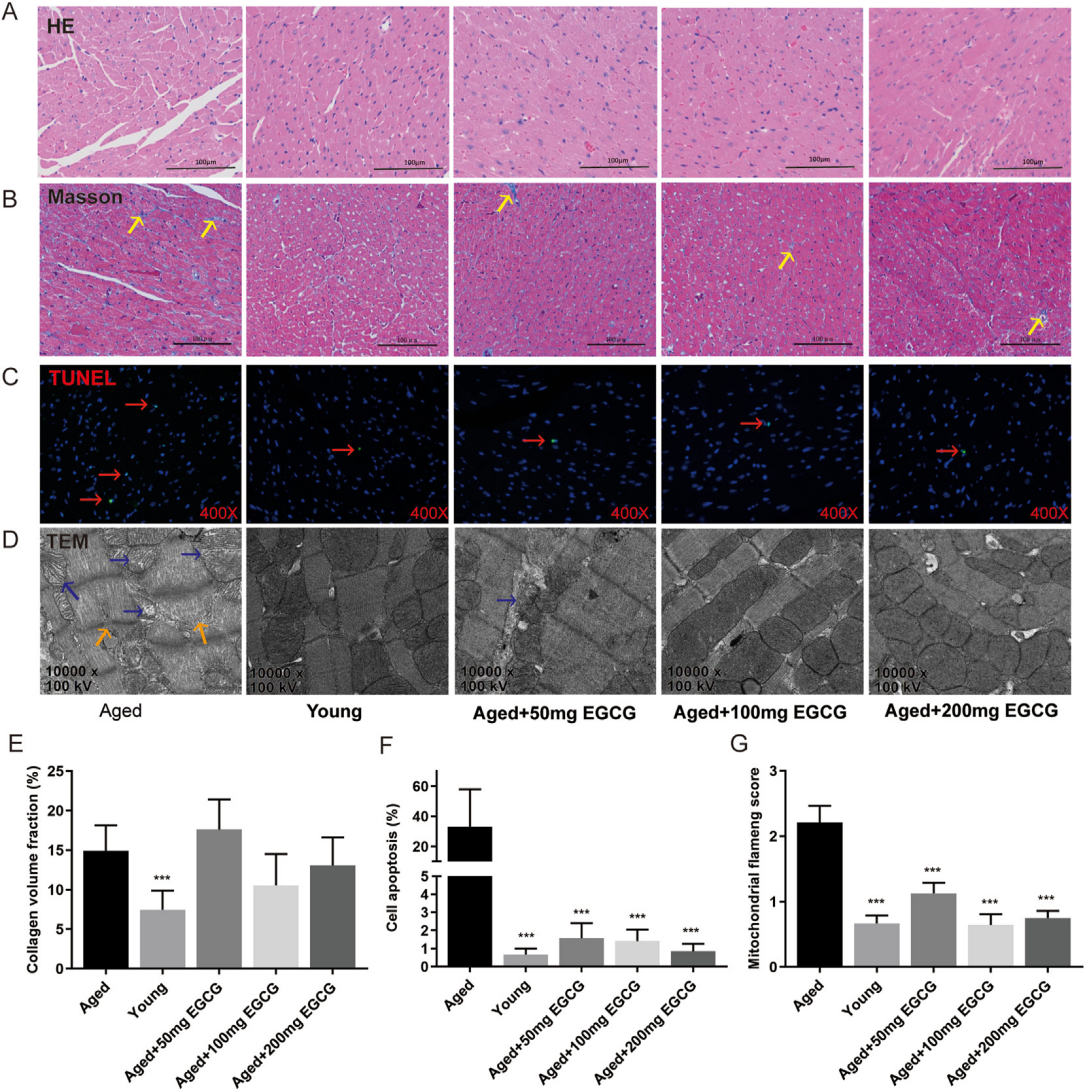
Long-term EGCG intake alleviates aging-related cardiomyocyte apoptosis and mitochondrial damage. **(A, B)** Representative histology of heart tissue sections stained with hematoxylin-eosin stain and Masson's trichrome stain (yellow arrows collagen fibrosis; scale bar: 100 μm). **(C)** TUNEL stain for evaluation of cardiomyocyte apoptosis (red arrows apoptotic cells; 400 × ). **(D)** Transmission electron microscope (TEM) for the display of myocardial ultrastructure (blue arrows damaged mitochondria and orange arrows twisted myofilaments; 10000 × ). **(E–G)** Quantitative analysis of myocardial fibrosis, cardiomyocyte apoptosis, and mitochondrial destruction (*n* = 3–4). All results are represented as mean ± standard deviation. ^∗∗∗^*P* < 0.001.

As the heart aging, cardiac structural and morphological changes became evident (Fig. 3). Heart tissue sections were stained with HE, Masson, and TUNEL to detect the degrees of myocardial fibrosis and cardiomyocyte apoptosis, and evaluate the effects of EGCG on the aged mice. And ultrastructure of the myocardium was also observed under TEM. Our data disclosed that heart aging was accompanied by cardiac fibrosis with a markedly increased interstitial fibrotic area (the young group *vs*. the aged group, *P* < 0.001; Fig. 3B, E), however, no significant inhibitory effect of all EGCG doses on myocardial fibrosis was observed in the aged mice (each of EGCG groups *vs*. the aged group, *P* > 0.05; Fig. 3A, B, E). There was a slight decrease in CVF in the aged mice treated with 100 and 200 mg EGCG, but the difference was not statistically significant. Intriguingly, a remarkable increase of cardiomyocyte apoptosis rate in the aged mice was revealed by TUNEL staining, as opposed to the young mice (*P* < 0.001; Fig. 3C, F), but it was normally reduced to the level of youth in the aged mice treated at all EGCG doses (each of EGCG groups *vs*. the aged group, *P* < 0.001; Fig. 3C, F), suggesting EGCG could prevent myocardial apoptosis in the aged heart. Meanwhile, the TEM showed that the sarcomeres were arranged in complex patterns and partially dissolved in the aged mice, accompanied by disrupted Z lines and an increased mitochondrial flameng score compared to the young mice (*P* < 0.001; Fig. 3D, G). Nevertheless, destruction of myofilaments and swollen mitochondria were alleviated in the aged mice administered with EGCG supplementations at all doses (each of the EGCG groups *vs*. the aged group: *P* < 0.001; Fig. 3D, G).

### Long-term administration of EGCG promoted cTnI expression via enhancing AcH3K9 in the aged mice

**Figure 4:**
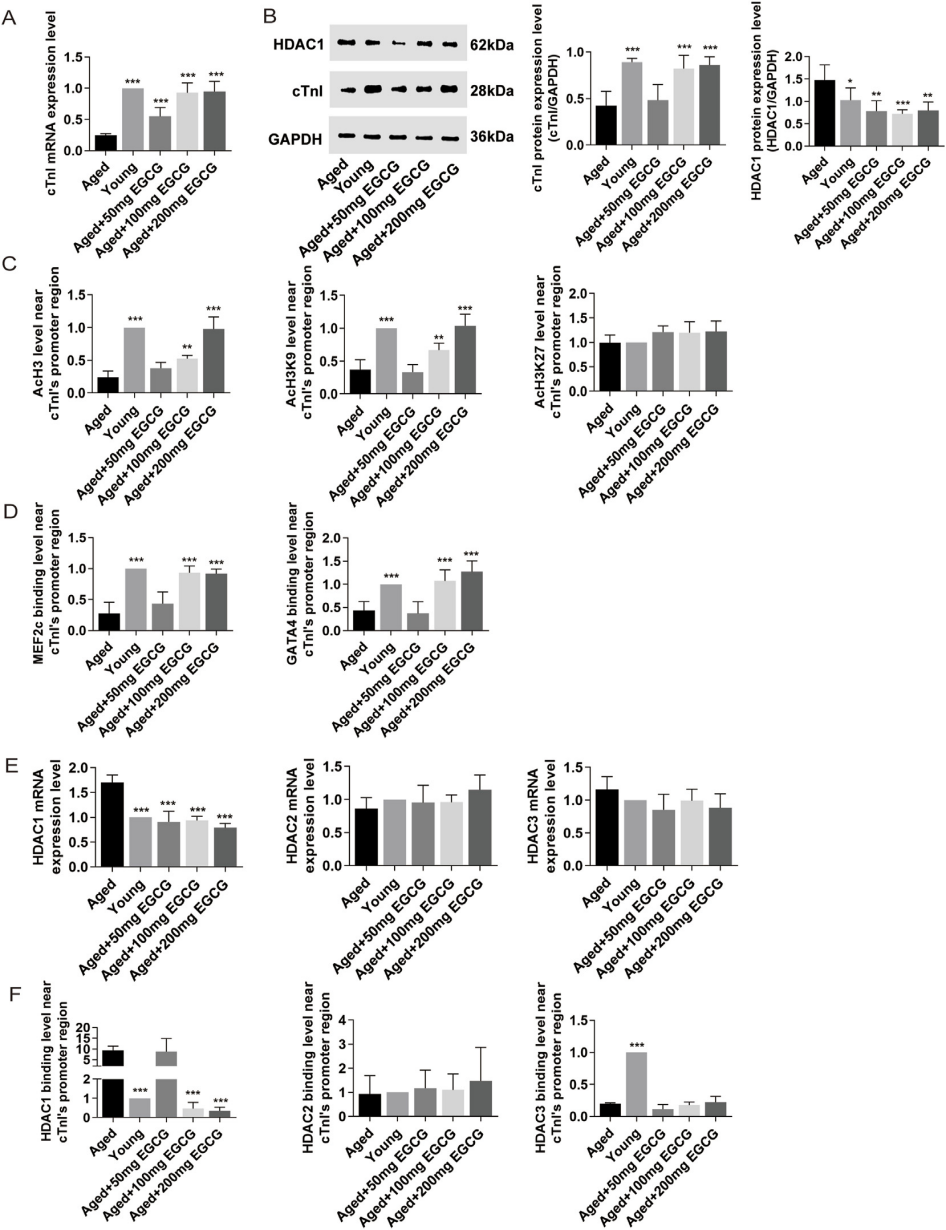
Long-term administration of EGCG promoted cTnI expression via inhibiting HDAC1 and enhancing AcH3K9. **(A, B)** Quantitative analysis of cTnI transcription and protein expression by quantitative PCR and Western blot, respectively. **(C)** The enrichment levels of AcH3K4, AcH3K9, and AcH3K27 near cTnI's promoter by chromatin immunoprecipitation (ChIP) assays. **(D)** The enrichment levels of MEF2c and GATA4 near cTnI's promoter by ChIP assays. **(E)** Quantitative analysis of class I HDACs by quantitative PCR. **(F)** The binding levels of class I HDACs near cTnI's promoter by ChIP assays (*n* = 8–10). All results are represented as mean ± standard deviation. ^∗^*P* < 0.05, ^∗∗^*P* < 0.01, ^∗∗∗^*P* < 0.001.

Abnormal cTnI expression, as reflected by its deficiency, low levels, and mutations has been associated with CDD.^9^^,^^10^ Emerging evidence revealed gene expression is consistent with histone acetylation modification.^10^^,^^16^ In this study, we detected the levels of cTnI expression and histone acetylation modification near its promoter both in young and aged mice with or without EGCG, to identify the impacts of EGCG on cTnI regulation. The cTnI mRNA and protein levels were significantly lower in the aged mice compared to the young mice (*P* < 0.001; Fig. 4A, B). Supplementation with 100 and 200 mg EGCG increased these reduced levels to those observed in the youth, however, 50 mg EGCG supplementation did not alter the protein decline (100 or 200 mg EGCG *vs*. the aged group: *P* < 0.001 for both; 50 mg EGCG *vs*. the aged group: *P* > 0.05; Fig. 4A, B). Furthermore, a significantly declined AcH3 near cTnI's promoter (*P* < 0.001) was found in the aged group, along with decreased AcH3K9 (*P* < 0.001) and unchanged AcH3K27 (*P* > 0.05) compared with the young mice (Fig. 4C). Supplementations with 100 and 200 mg EGCG notably increased the downregulated AcH3 and AcH3K9 near cTnI's promoter (100 or 200 mg EGCG *vs*. the aged group: *P* < 0.01 for both; 200 mg EGCG *vs*. the young group: *P* > 0.05) and did not affect AcH3K27 (each of EGCG groups *vs*. the aged group: *P* > 0.05). However, no significant improvement of AcH3 and AcH3K9 was found after 50 mg EGCG intake (50 mg EGCG *vs*. the aged group: *P* > 0.05 for both; Fig. 4C). In addition, the lower transcription factor binding levels of MEF2c and GATA4 near cTnI's promoter were confirmed in the aged mice (the young mice *vs*. the aged mice: *P* < 0.001; Fig. 4D) and improved to the levels as seen in the young mice by 100 and 200 mg EGCG administrations (100 or 200 mg EGCG *vs*. the aged group: *P* < 0.001 for both; 100 or 200 mg EGCG *vs*. the young group: *P* > 0.05 for both; Fig. 4D). The results indicated that EGCG could increase cTnI expression via enhancing AcH3 and AcH3K9 near cTnI's promoter and increasing the recruitment of MEF2c and GATA4 in the aged mice.

### Long-term administration of EGCG inhibited HDAC1 in the aged mice

To better understand the target of EGCG, we detected Class I HDACs, the upstream modifier of AcH3K9 in the heart tissues. The data showed the transcript level of HDAC1 was significantly higher in the aged mice than in the young mice (*P* < 0.001), and there was no difference in HDAC2 and HDAC3 between the two groups (*P* > 0.05 for both; Fig. 4E). The elevated protein level of HDAC1 was consistent with its transcription level (*P* < 0.001; Fig. 4B). But the boosted transcript and protein levels of HDAC1 were markedly inhibited by all doses of EGCG (each of EGCG groups *vs*. the aged group: *P* < 0.001 for transcript and *P* < 0.01 for protein; Fig. 4B, E). Furthermore, the HDAC1 binding level near cTnI's promoter was significantly higher in the aged mice than in the young mice (*P* < 0.001), but 100 and 200 mg EGCG supplementations significantly inhibited the elevated HDAC1 binding level (100 or 200 mg EGCG *vs*. the aged group: *P* < 0.001 for both; 100 or 200 mg EGCG *vs*. the young group: *P* > 0.05 for both; Fig. 4F) and had no effects on HDAC2 and HDAC3 (each of EGCG groups *vs*. the aged group: *P* > 0.05 for both; Fig. 4F). These results suggested that EGCG might enhance AcH3 and AcH3K9 near cTnI's promoter by inhibiting HDAC1.

### EGCG enhanced cTnI expression via inhibiting HDAC1

**Figure 5:**
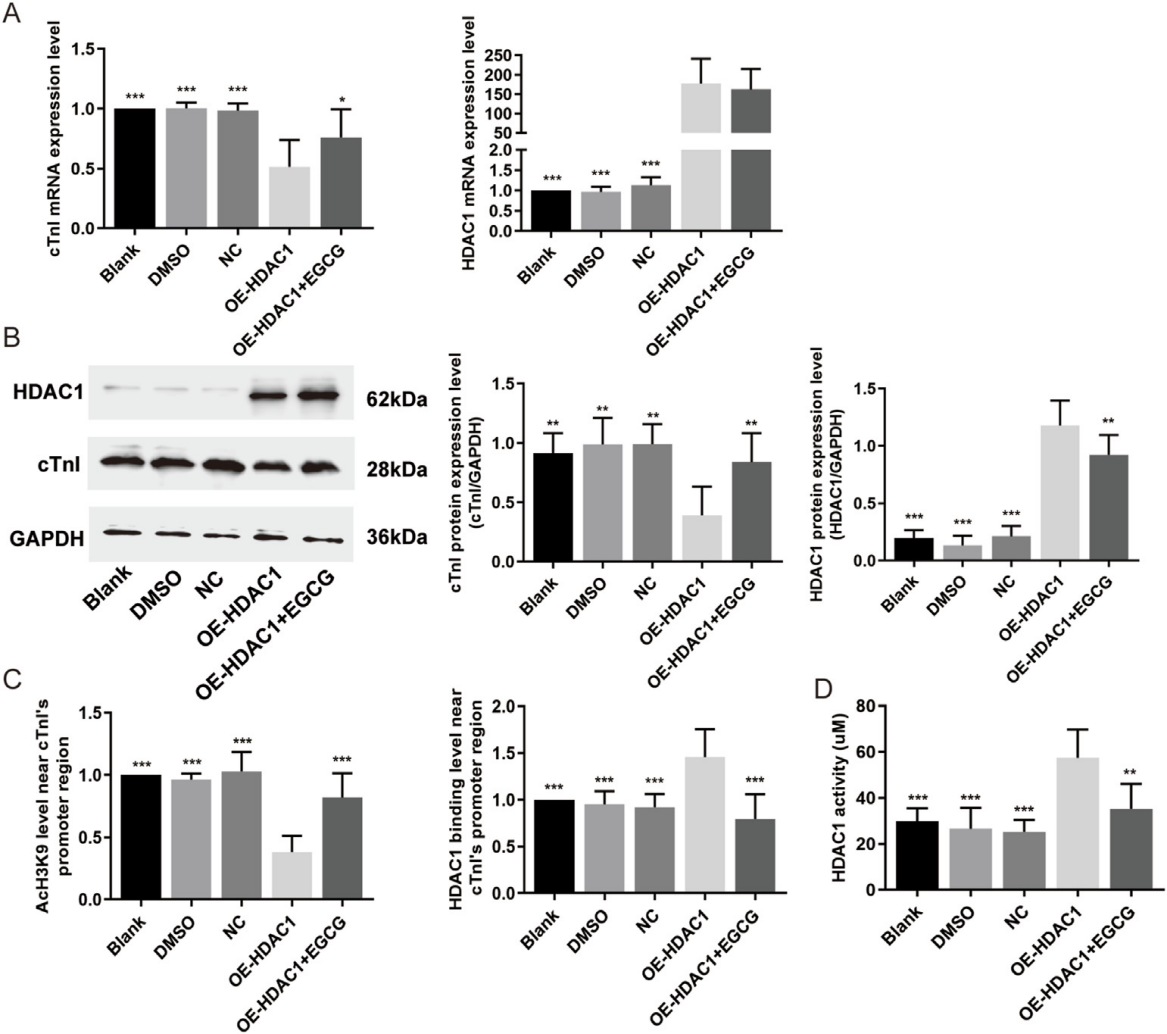
EGCG elevates cTnI expression via suppressing HDAC1 and increasing AcH3K9. **(A)** Quantitative analysis of cTnI and HDAC1 transcription expression by quantitative PCR. **(B)** Quantitative analysis of cTnI and HDAC1 protein expression by Western blot. **(C)** The enrichment levels of AcH3K9 and HDAC1 near cTnI's promoter by chromatin immunoprecipitation assays. **(D)** HDAC1 activity. All results are represented as mean ± standard deviation. ^∗^*P* < 0.05, ^∗∗^*P* < 0.01, ^∗∗∗^*P* < 0.001.

To directly confirm the regulatory effects of HDAC1 on cTnI expression, primary cardiomyocytes transfected with HDAC1 overexpression adenovirus were cultured *in vitro*. As shown in Figure 5A and B, cTnI mRNA and protein expression levels were significantly reduced in the HDAC1 overexpression group compared with the Blank group (*P* < 0.001 for transcript and *P* < 0.01 for protein). The transcription and protein levels for HDAC1, the enrichment of HDAC1 near cTnI's promoter, and its activity in cardiomyocytes were elevated in the HDAC1 overexpression group compared with the Blank group, along with decreased AcH3K9 (*P* < 0.001 for all; Fig. 5A–C). These data indicated that HDAC1 induced-histone deacetylation modification played a key role in low cTnI expression regulation.

**Figure 6:**
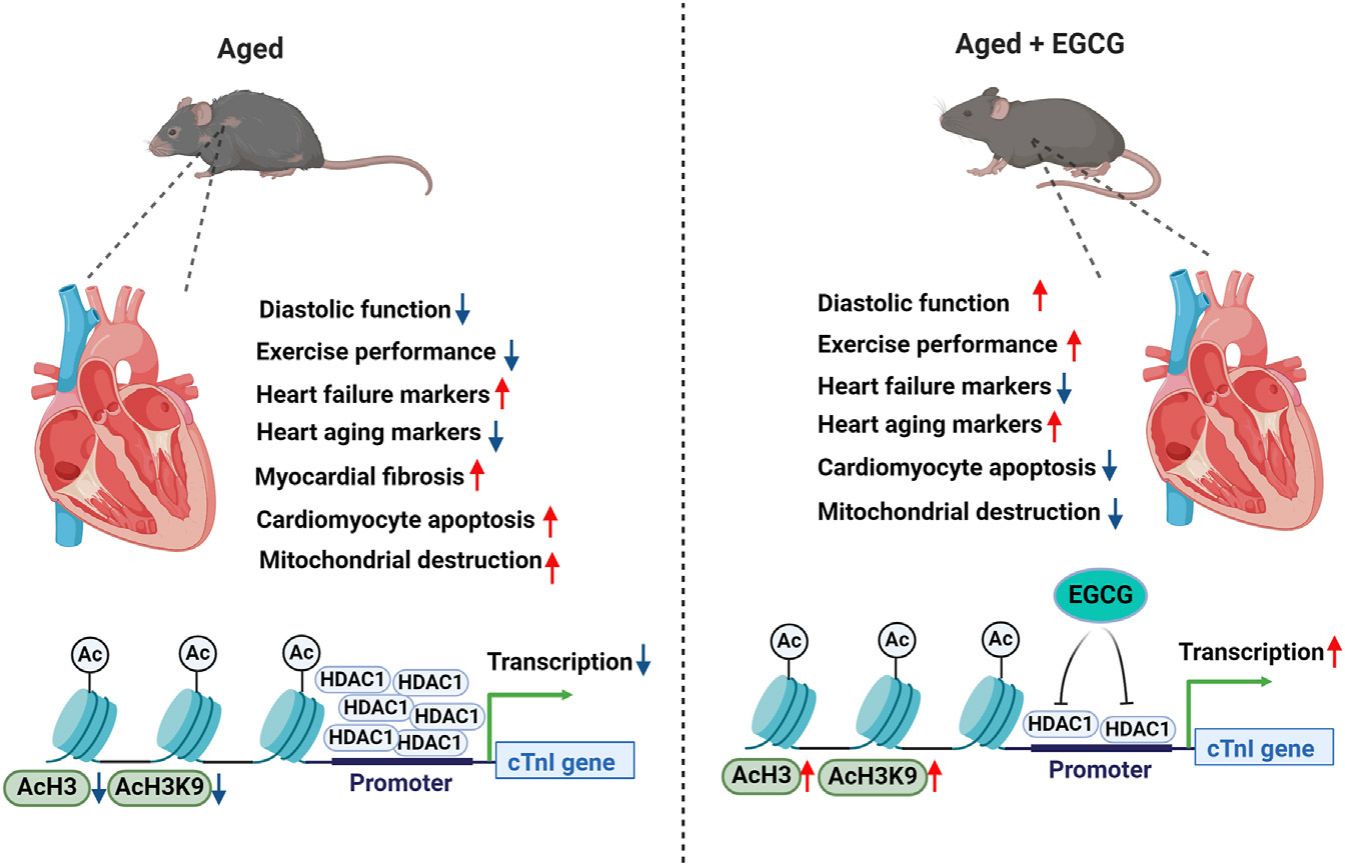
The schematic illustrating that long-term EGCG administration prevents CDD and cTnI decline in aging hearts. The scheme depicts the role of physiological aging in the pathophysiology of CDD. The hallmarks of HFpEF such as CDD, reduced exercise performance, increased heart failure markers, myocardial fibrosis, cardiomyocyte apoptosis, and mitochondrial destruction, which might be associated with cTnI decline induced by HDAC1. According to our results, long-term EGCG administration counteracts aging-associated cardiomyocyte apoptosis and mitochondrial destruction, through which it improves the physiological cardiac function and structure. In the aging mice, EGCG regulates cTnI gene expression via inhibiting the expression of HDAC1, reducing its binding level near cTnI's promoter region and lowering the activity, as well as enhancing AcH3 and AcH3K9. The black blue arrow indicates lower and the red arrow indicates higher. See text for details. AcH3, acetylated histone 3; AcH3K9, acetylated lysine 9 on histone H3; CDD, cardiac diastolic dysfunction; cTnI, cardiac troponin I; EGCG, epigallocatechin gallate; HDAC1, histone deacetylase 1; HFpEF, heart failure with preserved ejection fraction.

To better understand the effects of EGCG, primary cardiomyocytes were treated with 40 μM EGCG in the HDAC1 overexpression group (Fig. 5). The results showed the transcription and protein levels for cTnI were increased by EGCG treatment in cardiomyocytes with HDAC1 overexpression (*P* < 0.05 for transcript and *P* < 0.01 for protein). We then detected the histone acetylation modification levels. The results revealed the protein for HDAC1 and its enrichment near cTnI's promoter were significantly reduced (*P* < 0.001 for both), and the subsequent improvement of AcH3K9 was detected in HDAC1 overexpression group with EGCG administration (*P* < 0.001), accompanied by depressed HDAC1 activity (*P* < 0.01). Nevertheless, EGCG had no inhibitory effects on the mRNA expression of HDAC1 in primary cardiomyocytes with overexpressed HDAC1 (*P* > 0.05). These data confirmed that EGCG could enhance cTnI expression via inhibiting the HDAC1 protein expression, its enrichment near cTnI's promoter and the activity of HDAC1, and then elevating AcH3 and AcH3K9, as shown in Figure 6.

## Discussion

In recent years, EGCG, the major polyphenol component of green tea extract, has been widely reported to have extensive biological activities in various organs.^21^, ^22^, ^23^ In our study, we found that cTnI expression and diastolic function were decreased gradually during cardiac aging, therefore, EGCG was administered in drinking water before their declines occurred. Then the effects of different concentrations of EGCG were observed. The results showed that 100 and 200 mg EGCG supplementations in drinking water for 6 months prevented the development of aging-related CDD and cTnI expression decline. This effect was due to long-term intervention with 100 and 200 mg EGCG up-regulating cTnI expression via inhibiting HDAC1 and increasing AcH3K9 near cTnI's promoter. However, there was no significant difference in the performance between the 50 mg EGCG group and the aged group. We speculated that the concentrations of EGCG or its derivatives in the blood circulation of mice administered with 50 mg EGCG were too low to generate the cardioprotective effects. In our previous study, intraperitoneal injection of 50 mg EGCG could improve CDD in restrictive cardiomyopathy (RCM) mice, indicating that a higher dose of oral EGCG may be necessary to avoid first-pass elimination.

CDD, a clinical syndrome associated with aging, is becoming increasingly prevalent. Recent studies have indicated that abnormal expression of cTnI contributes to the pathophysiology and development of CDD.^9^, ^10^, ^11^^,^^24^ Homozygous knock-in mice with cTnI R21C mutation developed hypertrophy after 12 months of age and exhibited abnormal diastolic function that is characterized by longer filling times and impaired relaxation.^24^ Mice carrying cTnI R193H mutation manifested bi-enlarged atria and severe CDD.^25^ A clinical study found that the myocardial cytoplasmic cTnI concentration was decreased in the elderly human hearts, which was consistent with the occurrence of CDD in the elderly population.^11^ In hemodialysis patients with pEF, serum cTnI levels were significantly associated with CDD and risk of mortality independent of echocardiographic variables and other biomarkers.^26^ Our data showed that cTnI mRNA and protein expression declined markedly at 14 and 16 months of age, respectively, with the lowest expression occurring at 18 months. In fact, histone acetylation is a dynamic and reversible process that regulates gene expression and is mediated by histone acetylase and HDACs.^27^^,^^28^ Our data demonstrated that the expression of HDAC1 and its enrichment near cTnI's promoter were increased in the aged mice, and the level of AcH3K9, a marker for gene activation, was reduced, indicating aging-related low cTnI expression was associated with HDAC1 induced deacetylation of H3K9 near cTnI's promoter. We wonder whether blocking cTnI decline could prevent the occurrence and development of CDD.

In Asia, especially in China, there is a tradition of drinking tea for thousands of years. EGCG has been reported to have multiple effects on cardiovascular diseases and could improve CDD in RCM mice and patients with abnormal cTnI.^15^^,^^25^ EGCG dissolved in drinking water was used in the present study. This feeding regimen is noninvasive and well tolerated by animals and mimics the daily consumption of green tea by an average adult human.^29^ Based on the daily water consumption and weight of mice, we calculated the mean dose to be about 50, 100, and 200 mg/kg/day.

Our data showed that long-term EGCG administration blunted aging-related CDD and exercise performance decline, accompanied by significant reductions of HF markers and increases of SERCA2a and SAR in the EGCG-treated aged mice, suggesting EGCG could prevent cardiac aging and improve HF. SAR, a luminal Ca^2+^ buffer protein, is located in the longitudinal sarcoplasmic reticulum of muscle and heart and regulates Ca^2+^ reuptake by interacting with SERCA and maintaining its stabilization.^30^^,^^31^ Senescent SAR knock-out mice exhibited typical systolic dysfunction due to the decreases in SERCA2a expression and activity.^30^ AAV/SERCA2a gene therapy has been performed in patients with advanced heart failure due to systolic dysfunction.^32^ However, in our study, no difference was found in myocardial contractility between the young and aged mice. Systolic dysfunction may not be a major issue in older mice and the population.^2^^,^^3^^,^^6^ With increasing age, hearts present undesirable structural and morphologic phenotypes, characterized by elevated collagen fiber deposition, myocardial apoptosis, and mitochondrial destruction. Here, we found that EGCG may attenuate myocardial apoptosis and mitochondrial destruction in the aged mice, but had no effects on cardiac fibrosis. EGCG could maintain the baseline mitochondrial function and integrity, which is essential for preventing myocardial apoptosis during aging.^33^ Taken together, long-term EGCG supplementation is associated with ameliorative diastolic function and a repressible aging process.

Epigenetically, EGCG has been classified as an HDAC inhibitor, which can repress Class I HDACs to enhance histone acetylation and increase the transcription of target genes.^34^^,^^35^ In this study, long-term 100 and 200 mg EGCG treatment could alleviate the decreases in cTnI transcript and protein levels. Enrichment of HDAC1 near cTnI's promoter was reduced, and AcH3 and AcH3K9 were elevated after EGCG treatment in the aged mice, along with elevated recruitments of transcription factors. Although 50 mg EGCG inhibited HDAC1 expression, it did not increase cTnI mRNA expression nor improve histone acetylation and enrichment of transcription factors near cTnI's promoter. To better understand the effect of EGCG on HDAC1, primary cardiomyocytes with HDAC1 overexpression were treated with EGCG. We found both cTnI mRNA and protein were decreased in cardiomyocytes with HDAC1 overexpression, however, EGCG could reverse cTnI expression. The level of AcH3K9 was increased, and protein expression of HDAC1 and its enrichment near cTnI's promoter were significantly reduced by EGCG treatment. Additionally, the activity of HDAC1 was also markedly suppressed. These data strongly suggested that EGCG could increase cTnI expression via inhibiting HDAC1.

Unfortunately, there were some limitations in the present study. EGCG blood concentrations were not measured due to a lack of corresponding devices. Water containing EGCG was freshly prepared daily, its blood concentration might be maintained at a certain level to ensure the effectiveness of EGCG. Here, we did not detect the methylation levels near cTnI's promoter. Our previous study revealed no significant changes in methylation levels in mice of different ages, and histone acetylation might be more important as an epigenetic regulator than gene methylation in regulating cTnI gene expression.^10^

## Conclusion

The primary finding of this study is that EGCG may be an effective agent for preventing aging-related CDD and cTnI expression decline by inhibiting HDAC1 and increasing AcH3 and AcH3K9 near cTnI's promoter in aged mice (Fig. 6). We provide a novel insight into the fact that long-term administration of EGCG is an effective strategy in preventing the onset and development of aging-related CDD and cTnI expression decline. Drinking green tea daily may improve diastolic function in elderly individuals and enhance their quality of life.

## Ethics approval

This study was approved by the Institutional Review Board of Children's Hospital of Chongqing Medical University. All procedures were performed in accordance with the Helsinki Declaration.

## Author contributions

JT designed the study. JJQ and ZLJ experimented and analyzed the data. JT and JJQ interpreted the results of the experiments. JJQ and LJL prepared the figures. JJQ and ZLJ drafted the manuscript. All authors read and approved the final manuscript.

## Conflict of interests

The authors declared no conflict of interests.

## Funding

This study was supported by grants from the Science and Technology Foundation of Chongqing, China (No. cstc2021jcyj-bshX0056), the Key Grant from the National Clinical Research Center for Child Health and Disorders (China) (No. NCRCCHD-2021-KP-01), the National Natural Science Foundation of China (No. 81974030), and the National Key Clinical Specialty (China).
