# Supplementary material for: The effect of long-term administration of green tea catechins on aging-related cardiac diastolic dysfunction and decline of troponin I
Source: Genes Dis. 2024 Apr 3;12(2):101284. doi: 10.1016/j.gendis.2024.101284 (PMC11699727; doi:10.1016/j.gendis.2024.101284)
Supplement: Multimedia component 1 [file mmc1.docx]

**Supplementary Table** **1. Real-time PCR primer sequences**

| Gene | Forward | Reverse |
| --- | --- | --- |
| cTnI | GCAGGTGAAGAAGGAGGACA | CGATATTCTTGCGCCAGTC |
| ANP | TGCCCTCTTGAAAAGCAAAC | GGAAGCTGTTGCAGCCTAGT |
| BNP | ATCTCCTGAAGGTGCTGTCC | TGCATCTTGAATTGCTCTGG |
| HDAC1 | ATGAGCTGCCCTACAACGAC | GACGCTGCTTGATCTTCTCC |
| HDAC2 | GCCAAGTCAGAACAACTCAGC | GTCCTCAAACAGGGAAGGTT |
| HDAC3 | TTGAAGATGCTGAACCATGC | TGGCCTGCTGTAGTTCTCCT |
| SERCA2a | CAGTAACAAAAGCCCCCTCA | AAGCCATCCCTCCAAACTCT |
| SAR | CCGTGAGTCCCAGATAAGGA | GGAGGTTCCGTGACATTGAT |
| β-actin | CACACCCGCCACCAGTTCG | GTCCTTCTGACCCATTCCCACC |

**Supplementary** **Table 2. ChIP primer sequence**

| Gene | Forward | Reverse |
| --- | --- | --- |
| cTnI | CCAACTGGAGCTTTGCACACG | GAGGACACTGAGATAAGGGGCG |

**Supplementary Table 3. Cardiac functions before administration (12 months)**

| Parameters | 3m | Blank | 50mg EGCG | 100mg EGCG | 200mg EGCG |
| --- | --- | --- | --- | --- | --- |
| IVSd (cm) | 0.077±0.003 | 0.089±0.007 | 0.087±0.005 | 0.085±0.005 | 0.082±0.013 |
| LVIDd (cm) | 0.225±0.035 | 0.269±0.055 | 0.278±0.031 | 0.265±0.038 | 0.258±0.050 |
| LVPWd (cm) | 0.068±0.006 | 0.098±0.032 | 0.095±0.021 | 0.076±0.036 | 0.0821±0.028 |
| LVIDs (cm) | 0.102±0.021 | 0.139±0.027 | 0.146±0.025 | 0.137±0.029 | 0.128±0.035 |
| EDV (ml) | 0.040±0.017 | 0.053±0.029 | 0.058±0.015 | 0.053±0.021 | 0.050±0.027 |
| ESV (ml) | 0.003±0.006 | 0.007±0.006 | 0.008±0.005 | 0.005±0.006 | 0.006±0.007 |
| EF (%) | 92.36±1.85 | 85.45±0.55 | 84.67±4.00 | 85.38±3.66 | 85.84±6.67 |
| FS (%) | 58.58±3.29 | 48.28±0.81 | 47.73±5.13 | 48.46±4.43 | 50.18±9.69 |
| SV (ml) | 0.040±0.017 | 0.047±0.023 | 0.048±0.015 | 0.040±0.016 | 0.043±0.022 |
| E/A | 1.546±0.453 | 1.824±0.631 | 1.869±0.587 | 1.886±0.286 | 1.754±0.473 |
| IVRT (ms) | 11.994±5.000 | 14.3±3.008 | 12.399±3.005 | 10.148±2.061 | 10.669±3.248 |

A, late ventricular filling caused by atrial contraction; EDV, end-diastolic volume; EF, ejection fraction; ESV, end-systolic volume; FS, fractional shortening. IVSd, intraventricular septum at end-diastole; LVIDd, left ventricular internal dimensions at end-diastole; LVIDs, left ventricular internal dimensions at end-systole; LVPWd, left ventricular posterior wall thickness at end-diastole; All values are presented as mean ± SD. **P* ˂ 0.05, ***P* ˂ 0.01, ****P* ˂ 0.001; another four groups compared to 3m, respectively.
